# Beyond Physical Limitations: Depressive Mood and Self-Rated Health Among Adults with Severe Disabilities

**DOI:** 10.3390/healthcare14070916

**Published:** 2026-04-01

**Authors:** Hyun Namgung, Moon-June Oh

**Affiliations:** 1 Dharma College, Dongguk University, Seoul 04620, Republic of Korea; 2 Department of Policy Research, Seoul Welfare Foundation, Seoul 04147, Republic of Korea

**Keywords:** self-rated health, severe disability, depressive mood, functional capacity, IADL, unmet medical need, South Korea, Seoul

## Abstract

**Highlights:**

**What are the main findings?**

- Depressive mood was independently associated with self-rated health among adults with severe disabilities, after accounting for illness status and functional capacity.
- Living alone and unmet medical need were not significantly associated with self-rated health in the fully adjusted model.

**What are the implications of the main findings?**

- Alongside chronic disease management and functional support, attention to depressive mood and related psychosocial factors may be important for understanding perceived health among adults with severe disabilities.
- Attention to depressive mood and related psychosocial factors may be relevant to disability-related assessment and service planning, particularly for identifying individuals at risk of poorer perceived health.

**Abstract:**

**Background/Objectives**: Self-rated health (SRH) is a widely used summary indicator of health and a predictor of subsequent morbidity and mortality. Among adults with severe disabilities, SRH may reflect not only chronic conditions and functional limitations but also psychological factors, particularly depressive mood. This study examined the incremental contribution of depressive mood beyond physical and functional factors to SRH among adults with severe disabilities. **Methods**: We analyzed data from a survey of adults with severe disabilities in Seoul, South Korea (*N* = 1519). SRH (higher scores indicating better health) was modeled using block-wise hierarchical linear regression with robust standard errors. Models sequentially adjusted for (1) sociodemographic factors (including living arrangement); (2) disability characteristics (disability type and multiple disability status); (3) physical and functional health factors (illness status, instrumental activities of daily living (IADL), and unmet medical need); and (4) depressive mood. **Results**: In the fully adjusted model (*R*^2^ = 0.241), illness status (*b* = −0.330, *p* < 0.001), functional capacity (IADL; *b* = 0.116, *p* < 0.001), and depressive mood (*b* = −0.105, *p* < 0.001) were independently associated with SRH. Adding disability characteristics significantly improved model fit (Δ*R*^2^ = 0.074; Wald block *F*(3, 1510) = 42.56, *p* < 0.001). Further adding illness status, IADL, and unmet medical need improved model fit (Δ*R*^2^ = 0.051; Wald block *F*(3, 1507) = 30.85, *p* < 0.001), and depressive mood provided additional explanatory power (Δ*R*^2^ = 0.011; Wald block *F*(1, 1506) = 16.86, *p* < 0.001). Living alone and unmet medical need were not significantly associated with SRH after adjustment. **Conclusions**: Depressive mood was independently associated with SRH among adults with severe disabilities, even after accounting for physical health and functional limitations. These findings suggest that attention to depressive mood may be relevant to disability-related assessment and service planning, alongside chronic disease management and functional support. The observed association reflects a short-term affective state rather than clinical depression and should be interpreted within the context of subjective health appraisal.

## 1. Introduction

Self-rated health (SRH) is widely used as a global indicator of health status and has repeatedly demonstrated predictive validity for subsequent morbidity and mortality, even after accounting for objective clinical conditions and conventional risk factors [1,2,3]. Conceptually, SRH reflects an integrative appraisal process in which individuals synthesize bodily sensations, functional capacity, and psychosocial experiences within a given social and cultural context [4]. This multidimensional perspective is consistent with the World Health Organization’s International Classification of Functioning, Disability and Health (ICF) framework, which conceptualizes health and disability as the dynamic interaction between body functions, activities, participation, and contextual factors [5]. Nevertheless, disability-related health research and service delivery have often been dominated by a medical model that prioritizes somatic burden and functional limitations, potentially underemphasizing psychosocial determinants that shape perceived health [6,7,8]. Although both foundational and recent studies have highlighted the role of psychosocial and contextual factors in shaping self-rated health—particularly among individuals with chronic conditions and disabilities [9,10,11,12]—evidence remains limited on their incremental contribution to SRH after rigorously accounting for physical and functional health, particularly among adults with severe disabilities. Similar patterns have also been reported in international studies, where psychosocial, socioeconomic, and emotional factors were found to be associated with self-rated health across diverse populations and settings [11,12].

Notably, objective impairment severity does not always align with subjective health evaluations. The “disability paradox”—the observation that some individuals with serious disabilities report good quality of life—suggests that subjective assessments are not mechanically determined by physical constraints [13]. Related conceptual work on adaptation and response shift further indicates that individuals may recalibrate internal standards, values, or conceptualizations when living with chronic conditions [14,15]. Furthermore, evaluations of the same health states often differ systematically between patients and the general public [16]. In this broader context, psychosocial factors—particularly self-reported depressive mood referring to the day prior to the survey—may function as a salient “appraisal filter,” influencing symptom salience, perceived control, and coping resources [17,18,19]. Although short-term depressive mood states have been associated with poorer SRH in broader populations [20], the independent and incremental contribution of depressive mood to SRH among adults with severe disabilities remains underexamined, especially in analyses that rigorously account for chronic illness and functional impairment. Importantly, this study does not treat depressive mood as a proxy for clinical depression, but rather as a short-term affective state that may shape subjective health appraisal at the time of reporting.

This question is particularly relevant in Seoul, South Korea, where rapid population aging and the growing number of adults living with severe disabilities have heightened the need for integrated community-based services [21,22,23]. Adults with severe disabilities often face substantial health and functioning burdens, including chronic conditions and limitations in daily living [22,24]. They may also encounter social and structural barriers, such as accessibility challenges (e.g., mobility difficulties and longer travel times to service facilities) [25]. In addition, broader social determinants of health—including limited social support, stigma, and structural inequalities—may adversely affect psychosocial well-being and subjective health perceptions [26]. Because perceived health is shaped not only by objective limitations but also by psychosocial appraisal processes, gaps in identifying and addressing psychosocial needs, including depressive mood, may directly affect how individuals evaluate their overall health [17,18,19]. In settings where service systems primarily emphasize medical management and rehabilitation, psychosocial dimensions of health may be less systematically identified and addressed [27]. This gap underscores the importance of clarifying the distinct role of depressive mood in perceived health within this population, ultimately informing the development of more integrated community-based supports [28].

Therefore, the present study aimed to identify the determinants of SRH among community-dwelling adults with severe disabilities in Seoul. Based on prior literature on psychosocial appraisal processes, we hypothesized that depressive mood would be negatively associated with SRH even after accounting for sociodemographic characteristics, disability-related factors, and physical health conditions. In addition, we expected that depressive mood would provide incremental explanatory value beyond these established determinants.

While the incremental contribution of depressive mood may be modest, even small increases in explained variance can be meaningful in the context of SRH, which is shaped by multiple interacting determinants. By isolating the independent contribution of depressive mood, this study aimed to provide empirical evidence for integrating psychosocial considerations into disability-related health assessment and service delivery in Seoul and comparable metropolitan contexts.

## 2. Materials and Methods

### 2.1. Data and Study Population

This study analyzed data from the 2023 Survey on the Actual Conditions of Independent Living of People with Severe Disabilities in Seoul, conducted jointly by the Seoul Metropolitan Government and the Seoul Welfare Foundation. The survey assessed the living conditions and welfare needs of adults with severe disabilities residing in Seoul, South Korea.

The target population comprised community-dwelling adults aged 18 years or older with registered severe disabilities (formerly classified as disability grades 1–3 within the Korean disability registration system, which categorizes disability severity on a six-grade scale, with grades 1–3 corresponding to severe disability). Because severity classifications are determined separately for each disability type under this system, comparability of severity across disability categories may be limited. The sampling frame was constructed using the national disability registry maintained by the Ministry of Health and Welfare as of 31 December 2022. A stratified systematic sampling design was applied based on disability type, gender, and age to enhance representativeness. No additional exclusion criteria were applied beyond the predefined sampling frame and the use of complete-case data.

To reduce potential bias arising from underrepresentation of relatively small disability-type or age strata, the sampling design incorporated disproportionate allocation procedures, including minimum sample allocation within strata and square-root proportional allocation for selected strata. Post-stratification weights were subsequently constructed based on the inverse of the selection probability and further adjusted for nonresponse.

Data were collected between August and September 2023 through face-to-face interviews conducted by trained interviewers using Tablet-Assisted Personal Interviewing (TAPI), which incorporated real-time validation checks to reduce data entry errors. Although the initial target sample size was 1500 respondents, 1519 completed interviews were retained in the finalized dataset following field verification and data quality control procedures. The present study analyzed all eligible cases with complete information on the study variables (*N* = 1519).

Sampling weights were available in the original dataset. Given that the primary objective of this study was to estimate multivariable associations rather than population-level prevalence, the main analyses were conducted using unweighted regression models. Weighted regression models were additionally estimated as a sensitivity analysis to assess whether the substantive conclusions changed when sampling weights were applied. Robust standard errors were used to mitigate potential heteroskedasticity in the cross-sectional survey data.

Because descriptive statistics are presented for sample characterization, these estimates should be interpreted as sample-based rather than population-representative. Although the use of unweighted models may limit generalizability, prior research suggests that, when the goal is to estimate associations rather than population parameters, unweighted regression with appropriate covariate adjustment and robust standard errors can provide consistent estimates.

In accordance with the applicable national regulation, ethical review and approval were waived for this study because the data were collected by a public authority for the purpose of evaluating public welfare programs.

### 2.2. Measures

#### 2.2.1. Outcome Variable: Self-Rated Health (SRH)

Self-rated health (SRH) was assessed with a single item: “How would you rate your usual health status?” Responses were recorded on a 5-point Likert scale (1 = very good to 5 = very poor). For analysis, responses were reverse-coded so that higher scores indicated better perceived health (1 = very poor to 5 = very good). Single-item measures of SRH are widely used in population-based surveys and have demonstrated predictive validity for subsequent morbidity and mortality [1,2,3].

#### 2.2.2. Key Independent Variable: Depressive Mood

Depressive mood was assessed using a single self-reported item asking respondents, “To what extent did you feel depressed on the previous day?” Responses were rated on a 5-point Likert scale (1 = not at all to 5 = very much), with higher scores indicating greater depressive mood, and were treated as a continuous variable in the analysis. This measure captures a short-term affective state rather than clinical depression or a diagnosed depressive disorder. Although single-item measures have limitations in terms of reliability and depth, they are commonly used in large-scale population surveys and have been shown to reflect meaningful variations in subjective emotional states. In the context of self-rated health (SRH), which reflects an integrative and subjective appraisal of health status, momentary affective states such as depressive mood may function as an appraisal filter that shapes how individuals evaluate their overall health.

#### 2.2.3. Covariates

Sociodemographic covariates included gender (male = 1; female = 0), age (years), educational attainment, household income, and living arrangement. Educational attainment was coded on a six-level scale (1 = no formal education; 6 = graduate school or higher), entered as an ordinal score (1–6), and treated as a continuous covariate. Monthly household income (KRW) was natural log-transformed using ln(income + 1) to account for zero-income responses and reduce right skewness. Living arrangement was coded as a binary variable (1 = living alone; 0 = living with others).

Disability type was operationalized based on the primary registered disability type, using physical disability as the reference category. Physical disability comprised physical, brain lesion, facial, and internal organ disabilities (e.g., renal, cardiac, and respiratory impairments). Two dummy variables were created for sensory disability (visual, hearing, and speech disabilities) and mental disability (intellectual, autism spectrum, and psychiatric disabilities). In addition, multiple disability status was included as a separate binary indicator (1 = two or more disability types; 0 = a single disability type), capturing disability complexity independent of the primary disability classification.

Health-related covariates included illness status, functional capacity, and unmet medical need. Illness status was coded as 1 if the respondent reported a condition requiring medical diagnosis or treatment during the past six months and 0 otherwise. Functional capacity was assessed using an IADL index [29]. The index was computed as the mean of eight items (telephone use, shopping, meal preparation, housekeeping, laundry, medication management, financial management, and public transportation use). Each item was rated on a 4-point scale (1 = completely dependent; 4 = independent without assistance), with higher scores indicating greater functional independence. Unmet medical need was coded as 1 for respondents who reported an illness but did not receive necessary medical care during the past six months and 0 otherwise. Respondents reporting no illness were also coded as 0 for this variable.

### 2.3. Statistical Analysis

All statistical analyses were conducted using Stata/SE 17.0 (StataCorp LLC, College Station, TX, USA). Descriptive statistics were summarized as means with standard deviations (*SD*) for continuous variables and as frequencies with percentages for categorical variables. Because all study variables had complete data, all regression models were estimated using the full analytic sample (*N* = 1519).

To examine the association between depressive mood and self-rated health (SRH), we estimated block-wise hierarchical ordinary least squares (OLS) regression models with robust standard errors to account for potential heteroskedasticity in the cross-sectional survey data. SRH (range 1–5; higher scores indicating better health) was treated as continuous.

The use of OLS regression in a block-wise hierarchical framework was intended to facilitate the interpretation of incremental explanatory contributions (ΔR^2^) across conceptually grouped sets of variables. This approach was selected because the primary objective of the study was to compare the relative contribution of distinct predictor domains to SRH, rather than to model the ordinal distribution of the outcome itself.

Accordingly, predictors were entered sequentially in four blocks to assess their relative and incremental contributions to SRH. Block 1 included sociodemographic covariates (gender, age, education, log-transformed household income, and living arrangement). Block 2 added disability characteristics (sensory disability, mental disability, and multiple disability status). Block 3 additionally included physical and functional health covariates (illness status, IADL, and unmet medical need). Block 4 then entered the key independent variable, depressive mood.

The ordering of blocks was informed by a theoretical framework based on prior literature and the ICF model, reflecting a progression from more distal structural factors to more proximal health-related and affective factors. While this structure facilitates interpretation of incremental contributions, we acknowledge potential bidirectional relationships between physical and affective conditions; therefore, the block ordering should be interpreted as theory-driven rather than strictly causal.

Incremental explanatory power was evaluated using changes in *R*-squared (Δ*R*^2^) across blocks, and block-wise nested-model comparisons were conducted using Wald *F* tests under the robust variance estimator. Regression results are presented as unstandardized coefficients (*b*) with robust standard errors (SE), while 95% confidence intervals (CI) for selected key estimates from the fully adjusted model are reported in the Results text. Multicollinearity was assessed using variance inflation factors (*VIF*).

Although SRH and several predictors were measured on ordinal scales, they were treated as continuous variables in the main analysis. This approach is commonly adopted when Likert-type variables approximate underlying continuous constructs and when interpretability of effect sizes is prioritized. To assess the robustness of this specification, ordered logistic regression models with robust standard errors were estimated using the same covariate set, and the results were substantively consistent with those obtained from the OLS models (Table S1). In addition, weighted OLS regression models with robust standard errors were estimated using the provided sampling weights as a further sensitivity analysis, and these results were also substantively consistent with those from the main unweighted OLS models (Table S2).

## 3. Results

### 3.1. Participant Characteristics

Participant characteristics of the study population are presented in Table 1. The analytic sample included 1519 adults with severe disabilities. The mean age of the participants was 55.1 years (*SD* = 18.4). More than half of the respondents were male (58.9%), and the mean educational attainment score was 3.6 (*SD* = 1.2). The mean monthly household income was 220.3 (*SD* = 349.1) in units of 10,000 KRW, and 29.6% of respondents lived alone.

In terms of disability characteristics based on the primary registered disability type, physical disability was the most common type (54.5%), followed by mental (29.7%) and sensory disabilities (15.8%). Approximately 12.3% of participants reported having multiple disabilities.

Regarding health status, 69.7% of respondents reported experiencing an illness requiring medical diagnosis or treatment during the past six months. The mean IADL score was 2.92 (*SD* = 1.10). Among respondents reporting illness (*n* = 1059), 3.6% reported unmet medical need. The mean score for depressive mood was 2.89 (*SD* = 0.89), and the mean SRH score was 2.58 (*SD* = 0.90).

### 3.2. Block-Wise Hierarchical Regression Results for Self-Rated Health (SRH)

Table 2 summarizes the block-wise hierarchical OLS regression models for SRH. In Model 1 (sociodemographic characteristics), SRH was positively associated with male gender (*b* = 0.129, *p* < 0.01) and negatively associated with advancing age (*b* = −0.015, *p* < 0.001). Education, log-transformed household income, and living alone yielded no statistically significant associations with SRH at this stage. The model accounted for 10.5% of the variance in SRH (*R*^2^ = 0.105, adjusted *R*^2^ = 0.102).

In Model 2, the addition of disability characteristics significantly improved model explanatory power (Δ*R*^2^ = 0.074; Wald block *F*(3, 1510) = 42.56, *p* < 0.001). Compared with the physical disability reference group, both sensory disability (*b* = 0.446, *p* < 0.001) and mental disability (*b* = 0.473, *p* < 0.001) were associated with higher SRH, whereas multiple disability status was associated with lower SRH (*b* = −0.294, *p* < 0.001). Household income became statistically significant after adjustment for disability characteristics (*b* = 0.054, *p* < 0.05) and remained significant in the final model (*b* = 0.052, *p* < 0.05).

In Model 3, the inclusion of health and functioning variables further enhanced the model’s explanatory power (Δ*R*^2^ = 0.051; Wald block *F*(3, 1507) = 30.85, *p* < 0.001). Illness requiring diagnosis or treatment was inversely associated with SRH (*b* = −0.341, *p* < 0.001), while higher functional independence (IADL) showed a positive association (*b* = 0.122, *p* < 0.001). Unmet medical need remained non-significant. Following the inclusion of these variables, the coefficients for sensory disability (from *b* = 0.446 to *b* = 0.389) and multiple disabilities (from *b* = −0.294 to *b* = −0.180) were attenuated, while remaining statistically significant.

In the final fully adjusted model (Model 4; *R*^2^ = 0.241, adjusted *R*^2^ = 0.235), depressive mood was entered as the key independent variable, contributing additional explanatory power (Δ*R*^2^ = 0.011; Wald block *F*(1, 1506) = 16.86, *p* < 0.001). Even after adjustment for all preceding blocks, depressive mood was inversely associated with SRH (*b* = −0.105, 95% CI [−0.155, −0.055], *p* < 0.001). The coefficients for illness (Model 3: *b* = −0.341; Model 4: *b* = −0.330) and IADL (Model 3: *b* = 0.122; Model 4: *b* = 0.116) changed minimally after adding depressive mood, consistent with its incremental contribution beyond physical and functional health factors. Compared with the physical disability reference group, disability type remained positively associated with SRH in Model 4 (sensory disability: *b* = 0.387, 95% CI [0.274, 0.501]; mental disability: *b* = 0.483, 95% CI [0.382, 0.583]; both *p* < 0.001), while multiple disabilities remained inversely associated with SRH (*b* = −0.192, 95% CI [−0.330, −0.054], *p* < 0.01). Living alone (*b* = −0.033, 95% CI [−0.124, 0.059]) and unmet medical need (*b* = −0.053, 95% CI [−0.270, 0.164]) were not significantly associated with SRH.

Overall, lower SRH was associated with older age, multiple disabilities, recent illness, lower functional independence (IADL), and higher depressive mood, whereas sensory and mental disability types (relative to physical disability) and higher household income were associated with higher SRH.

Multicollinearity was minimal (mean *VIF* = 1.16; all *VIFs* ≤ 1.55), and the ordered logistic regression sensitivity analysis yielded substantively consistent findings (Supplementary Table S1). As an additional robustness check, weighted OLS regression models using the provided sampling weights also produced substantively consistent results, with no meaningful changes in the direction, magnitude, or statistical significance of the key coefficients (Supplementary Table S2).

## 4. Discussion

### 4.1. Principal Findings and Interpretation

This study examined the relative and incremental contributions of sociodemographic characteristics, disability characteristics, physical and functional health factors, and depressive mood to self-rated health (SRH) among adults with severe disabilities in Seoul. In the fully adjusted model (*R*^2^ = 0.241), illness status and functional capacity (IADL) were consistently associated with SRH, and depressive mood remained inversely associated with SRH after adjustment for all preceding blocks. Notably, depressive mood was inversely associated with SRH in the final model (*b* = −0.105, 95% *CI* [−0.155, −0.055]) and provided modest incremental explanatory power (Δ*R*^2^ = 0.011) beyond sociodemographic, disability, and physical/functional health factors. In practical terms, a one-point increase in depressive mood (on the 1–5 scale) corresponded to an average 0.105-point decrease in SRH (on the 1–5 scale), net of covariates. These findings were substantively consistent with those of the ordered logistic regression sensitivity analysis (Table S1). Although the incremental increase in explained variance (Δ*R*^2^ = 0.011) was modest, even small increments may be meaningful in the context of self-rated health, which reflects a multidimensional construct shaped by multiple interacting determinants. From a clinical and public health perspective, this finding may have particular relevance for screening and risk identification, rather than indicating a large standalone effect.

The observed association between depressive mood and SRH is consistent with prior evidence that SRH reflects an integrative appraisal of health that extends beyond somatic conditions to include psychological well-being [4]. More specifically, our findings align with prior studies showing that short-term affective states and emotional conditions are significantly associated with SRH even after accounting for physical health status [11,12]. Among adults with severe disabilities, depressive mood may be related to differences in overall health appraisal through multiple pathways, including reduced coping resources, diminished motivation for self-care, and increased salience of symptoms or functional difficulties [17,18,19]. Although the cross-sectional design precludes conclusions about temporality, the persistence of the depressive mood association after adjustment for illness status and functional capacity suggests that depressive mood captures a distinct dimension of perceived health rather than merely reflecting physical burden [30,31]. Importantly, the depressive mood measure used in this study reflects a short-term self-reported affective state experienced on the previous day rather than clinically diagnosed depression or a depressive disorder. Given that SRH represents a subjective appraisal of health status, such momentary affective states may systematically influence how individuals perceive and report their overall health, independent of underlying clinical conditions.

At the same time, these affective states may also reflect broader social and structural conditions. In the context of severe disability, social exclusion, limited participation, and structural barriers may plausibly contribute to depressive mood and influence how individuals appraise their health. This perspective is consistent with contemporary disability research emphasizing the role of social context and environmental constraints in shaping both psychological well-being and perceived health.

Physical and functional health factors showed robust associations with SRH. Illness requiring diagnosis or treatment in the past six months was inversely associated with SRH, while higher IADL scores—indicating greater functional independence—were positively associated with SRH [29]. These findings are consistent with prior studies demonstrating that both disease burden and functional capacity are core determinants of SRH across diverse populations [9,11]. These patterns underscore that SRH in this population is strongly shaped by both medical burden and the capacity to manage everyday activities, aligning with disability research emphasizing function as a core determinant of health-related quality of life [5,8].

Disability characteristics also contributed meaningfully to SRH. Multiple disability status remained inversely associated with SRH, supporting the notion that disability complexity may compound health-related challenges beyond primary impairment categories [32]. In contrast, sensory and mental disability categories showed higher SRH relative to the physical disability reference group. This pattern warrants cautious interpretation: while it may be compatible with the “disability paradox,” it may also reflect compositional heterogeneity within the reference group [13]. The physical disability category in this study encompassed a broad set of impairments, including internal organ disabilities, which may be accompanied by substantial comorbidity and symptom burden [33]. Accordingly, these differences may stem from variation in health profiles and functional burden within the physical disability reference group rather than inherently better health status in sensory or mental disability groups. Further research with more granular disability subtypes and comorbidity measures is warranted to clarify these between-group differences.

Two covariates—living alone and unmet medical need—were not significantly associated with SRH in the fully adjusted model. For living arrangement, a binary indicator may not adequately capture the quality of social support, caregiving resources, or community participation, which may be more proximal determinants of perceived health than co-residence status per se [34]. This interpretation is consistent with research suggesting that social ties and broader social integration, rather than living arrangement alone, are more closely related to subjective health outcomes [26,35]. For unmet medical need, the measure was defined conditional on illness and had a low prevalence, which may have limited statistical power and may also reflect heterogeneous reasons for unmet need (e.g., short delays vs. sustained barriers) [25,35]. These measurement features may partly explain the null associations.

### 4.2. Implications for Practice and Policy

From a practice and policy perspective, recent depressive mood may be relevant to disability-related assessment, particularly for identifying individuals at risk of poorer perceived health. These cross-sectional findings do not demonstrate intervention effects. However, they may still inform discussions of integrated service models linking medical rehabilitation, chronic disease management, functional support, and psychosocial or mental health support—particularly for individuals with multiple disabilities, who reported lower SRH (b = −0.192) [5,8]. In the context of Seoul, policies supporting independent living may also consider strengthening referral pathways between disability services and psychosocial or mental health support resources. Such efforts could include brief screening in community-based assessments and clearer follow-up pathways for individuals reporting elevated recent depressive mood [23,27]. In addition, the consistent association between functional capacity and SRH underscores the importance of considering daily functioning alongside psychosocial factors in disability-related assessment and service planning [5,8,28]. Particular attention may be warranted for subgroups with higher functional limitations and socioeconomic disadvantage, who may face compounded barriers to accessing integrated support. Taken together, these findings may inform coordinated approaches that consider medical conditions, functional needs, and psychosocial well-being together, in ways that may better reflect how individuals with severe disabilities appraise their overall health.

### 4.3. Strengths and Limitations

This study has several strengths. It used a large, population-based survey of adults with severe disabilities in Seoul and applied a block-wise hierarchical modeling strategy that enabled explicit evaluation of incremental contributions across domains. Robust standard errors were used to reduce sensitivity to heteroskedasticity, and results were checked using an ordered logistic regression sensitivity analysis.

Several limitations should be noted. First, the cross-sectional design limits causal interpretation and does not establish temporal ordering between depressive mood and SRH. Second, SRH and depressive mood were assessed using single items; specifically, the depressive mood item reflects recent affect (“yesterday”) and may not capture clinically validated depressive symptomatology or longer-term mental health status. This difference in reference periods (usual health vs. yesterday’s mood) may introduce reporting noise and could attenuate observed associations, as well as limit direct comparability with measures of clinical depression. Third, unmet medical need was operationalized conditional on illness and had a low prevalence, potentially limiting precision. Fourth, analyses were conducted without sampling weights because the analytic goal was to estimate multivariable associations rather than population prevalence; therefore, descriptive estimates should not be interpreted as population-level prevalence for all adults with disabilities in Seoul [36]. Finally, residual confounding may remain due to unmeasured contextual factors such as accessibility of the built environment, availability of informal caregiving, and broader social support.

### 4.4. Future Research

Future research should test these associations using longitudinal designs to clarify the directionality and potential reciprocal relationships among depressive mood, psychosocial well-being, and SRH. Studies incorporating validated multi-item measures of depressive symptoms and richer psychosocial indicators (e.g., perceived social support, social participation, stigma, and service accessibility) would help clarify mechanisms. Further analyses using more detailed disability subtypes and comorbidity profiles may also elucidate heterogeneity within disability categories and identify subgroups most likely to benefit from integrated interventions. Together, such work would strengthen the evidence base for designing and targeting integrated disability services that address medical, functional, and psychosocial needs.

## 5. Conclusions

Depressive mood was independently associated with self-rated health (SRH) among adults with severe disabilities in Seoul, after accounting for sociodemographic, disability, and physical and functional health factors. Although its incremental explanatory contribution was modest, depressive mood may still be relevant to disability-related assessment and service planning, alongside chronic disease management and functional support. A coordinated approach that considers medical burden, functional independence, and psychosocial well-being together may be better aligned with the multidimensional nature of perceived health in this population. Further longitudinal studies using validated multi-item measures are needed to clarify the directionality of these associations.

## Figures and Tables

**Table 1:** General characteristics of the study population (*N* = 1519).

Characteristics	Categories/Units	*N* (%) or Mean ± *SD*
**Sociodemographic Characteristics**		
Gender	Male	894 (58.9%)
	Female	625 (41.1%)
Age	Years	55.1 ± 18.4
Education	Score (1–6)	3.6 ± 1.2
Household income ^1^	Monthly (10,000 KRW)	220.3 ± 349.1
Living arrangement	Living alone	450 (29.6%)
	Living with others	1069 (70.4%)
**Disability characteristics**		
Disability Type	Physical Disability	828 (54.5%)
	Sensory Disability	240 (15.8%)
	Mental Disability	451 (29.7%)
Multiple Disabilities	Yes	187 (12.3%)
	No	1332 (87.7%)
**Health and Functioning**		
Illness (past 6 months)	Yes	1059 (69.7%)
	No	460 (30.3%)
IADL	Score (1–4)	2.92 ± 1.10
Unmet medical need ^2^	Yes	38 (3.6%)
	No	1021 (96.4%)
**Psychosocial Factor**		
Depressive mood	Score (1–5)	2.89 ± 0.89
**Outcome**		
Self-rated health (SRH)	Score (1–5)	2.58 ± 0.90

^1^ Household income is presented as raw monthly income before log-transformation. ^2^ Unmet medical need was assessed among respondents reporting illness (*n* = 1059).

**Table 2:** Block-wise hierarchical OLS regression results for self-rated health (SRH) (*N* = 1519).

Predictors	Model 1 (Sociodemographic)	Model 2 (+Disability Characteristics)	Model 3 (+Health and Functioning)	Model 4 (+Depressive Mood)
**Sociodemographic Characteristics**				
Gender (male)	0.129 ** (0.045)	0.142 ** (0.043)	0.125 ** (0.042)	0.120 ** (0.042)
Age (years)	−0.015 *** (0.001)	−0.011 *** (0.001)	−0.010 *** (0.001)	−0.010 *** (0.001)
Education (1–6)	−0.006 (0.021)	0.014 (0.021)	0.006 (0.020)	0.008 (0.020)
Household income ^1^	0.043 (0.024)	0.054 * (0.023)	0.057 ** (0.022)	0.052 * (0.022)
Living alone	−0.013 (0.047)	0.013 (0.046)	−0.044 (0.047)	−0.033 (0.047)
**Disability characteristics**				
Sensory disability		0.446 *** (0.060)	0.389 *** (0.059)	0.387 *** (0.058)
Mental disability		0.473 *** (0.052)	0.478 *** (0.051)	0.483 *** (0.051)
Multiple disabilities		−0.294 *** (0.071)	−0.180 * (0.070)	−0.192 ** (0.070)
**Health and functioning**				
Illness (past 6 months)			−0.341 *** (0.046)	−0.330 *** (0.046)
IADL (1–4)			0.122 *** (0.021)	0.116 *** (0.021)
Unmet medical need ^2^			−0.071 (0.110)	−0.053 (0.111)
**Psychosocial Factor**				
Depressive mood (1–5)				−0.105 *** (0.026)
Constant	3.123 *** (0.180)	2.613 *** (0.187)	2.462 *** (0.184)	2.783 *** (0.195)
**Model fit statistics**				
*R* ^2^	0.105	0.179	0.230	0.241
Adj. *R*^2^	0.102	0.175	0.225	0.235
Δ*R*^2^	-	0.074	0.051	0.011
Wald block *F* (df1, df2)	-	42.56 (3, 1510)	30.85 (3, 1507)	16.86 (1, 1506)

Notes: Entries are unstandardized coefficients (*b*) with robust *SE* in parentheses. Physical disability is the reference category. Δ*R*^2^ indicates the change in *R*^2^ from the previous model; block effects were tested using robust Wald *F* tests. * *p* < 0.05, ** *p* < 0.01, *** *p* < 0.001. ^1^ Household income was modeled as ln(income + 1). ^2^ Unmet medical need was assessed among respondents reporting illness (*n* = 1059).

## Data Availability

Restrictions apply to the availability of these data. Data were obtained from the Seoul Welfare Foundation and are available with the permission of the Seoul Welfare Foundation due to privacy and institutional restrictions. The analytic dataset containing the variables used in this study, together with the associated analysis code (e.g., Stata .do files), is available from the corresponding author upon reasonable request, subject to institutional and data protection requirements.

## Supplementary Materials

The following supporting information can be downloaded at: https://www.mdpi.com/article/10.3390/healthcare14070916/s1, Table S1. Sensitivity Analysis Using Ordered Logistic Regression for Self-Rated Health (SRH) (*N* = 1519). Table S2. Sensitivity Analysis Using Weighted OLS Regression for Self-Rated Health (SRH) (*N* = 1519).

## Abbreviations

The following abbreviations are used in this manuscript:

SRH	Self-Rated Health
IADL	Instrumental Activities of Daily Living
TAPI	Tablet-Assisted Personal Interviewing
