# Supplementary material for: Beyond Physical Limitations: Depressive Mood and Self-Rated Health Among Adults with Severe Disabilities
Source: Healthcare (Basel). 2026 Apr 1;14(7):916. doi: 10.3390/healthcare14070916 (PMC13072908; doi:10.3390/healthcare14070916)
Supplement: Supplementary file 1 [file healthcare-14-00916-s001.zip › healthcare-4191872-supplementary.pdf]

Supplementary Materials

Table S1. Sensitivity Analysis Using Ordered Logistic Regression for Self-Rated Health (SRH) (*N* = 1,519)

Predictors	OR	Robust *SE*	*p*-value	95% CI
**Sociodemographic Characteristics**				
Gender (male)	1.344	0.134	0.003	1.106, 1.634
Age (years)	0.976	0.003	< 0.001	0.970, 0.983
Education (1–6)	1.009	0.047	0.856	0.920, 1.106
Household income (log)	1.155	0.059	0.005	1.045, 1.276
Living alone	0.949	0.104	0.631	0.766, 1.175
**Disability characteristics**				
Sensory disability	2.527	0.353	< 0.001	1.922, 3.323
Mental disability	3.187	0.391	< 0.001	2.506, 4.052
Multiple disabilities	0.630	0.112	0.009	0.445, 0.892
**Health and functioning**				
Illness (past 6 months)	0.462	0.050	< 0.001	0.374, 0.570
IADL (1–4)	1.345	0.070	< 0.001	1.214, 1.489
Unmet medical need	0.917	0.243	0.745	0.546, 1.542
**Psychosocial Factor**				
Depressive mood (1–5)	0.760	0.048	< 0.001	0.670, 0.861

* Model statistics: Wald χ^2 (12) = 388.05 ($p < 0.001$); Log pseudolikelihood = −1766.52; Pseudo R^2 = 0.109.

Notes: Ordered logistic regression with robust standard errors. SRH was coded so that higher values indicate better health; therefore, OR > 1 indicates higher odds of reporting better SRH. Physical disability was the reference category for disability type. Cutpoints are omitted for brevity.

Table S2. Sensitivity Analysis Using Weighted OLS Regression for Self-Rated Health (SRH) (*N* = 1,519)

Predictors	*b*	Robust *SE*	*p*-value	95% CI
**Sociodemographic Characteristics**				
Gender (male)	0.158	0.045	< 0.001	0.070, 0.246
Age (years)	−0.011	0.001	< 0.001	−0.014, −0.009
Education (1–6)	0.004	0.022	0.838	−0.038, 0.047
Household income (log)	0.037	0.027	0.176	−0.017, 0.090
Living alone	−0.082	0.051	0.106	−0.181, 0.017
**Disability characteristics**				
Sensory disability	0.408	0.063	< 0.001	0.284, 0.532
Mental disability	0.443	0.054	< 0.001	0.338, 0.548
Multiple disabilities	−0.257	0.076	0.001	−0.406, −0.107
**Health and functioning**				
Illness (past 6 months)	−0.322	0.049	< 0.001	−0.418, −0.226
IADL (1–4)	0.122	0.022	< 0.001	0.079, 0.166
Unmet medical need	−0.042	0.127	0.743	−0.290, 0.207
**Psychosocial Factor**				
Depressive mood (1–5)	−0.109	0.028	< 0.001	−0.164, −0.054

* Model statistics: F (12, 1506) = 44.97, p < 0.001; R^2 = 0.280.

Notes: Weighted ordinary least squares (OLS) regression with robust standard errors. Sampling weights were applied. Variable coding is identical to that used in the main analysis.
